# “Fast” Plasmons Propagating in Graphene Plasmonic Waveguides with Negative Index Metamaterial Claddings

**DOI:** 10.3390/nano10091637

**Published:** 2020-08-20

**Authors:** Zeyang Zhao, Shaojian Su, Hengjie Zhou, Weibin Qiu, Pingping Qiu, Qiang Kan

**Affiliations:** 1 College of Information, Science and Engineering, Huaqiao University, Xiamen 361021, China; zyzhao@stu.hqu.edu.cn (Z.Z.); hjzhou@stu.hqu.edu.cn (H.Z.); 2 Fujian Key Laboratory of Semiconductor Materials and Applications, Xiamen University, Xiamen 361005, China; 3 College of Materials Science and Opto-Electronic Technology, University of Chinese Academy of Sciences, Beijing 100086, China; ppqiu@semi.ac.cn (P.Q.); kanqiang@semi.ac.cn (Q.K.); 4 Institute of Semiconductors, Chinese Academy of Sciences, Beijing 100086, China

**Keywords:** negative refraction index, fast light, metamaterial, graphene core

## Abstract

We propose the monolayer graphene plasmonic waveguide (MGPW), which is composed of graphene core sandwiched by two graphene metamaterial (GMM) claddings and investigate the properties of plasmonic modes propagating in the waveguide. The effective refraction index of the GMMs claddings takes negative (or positive) at the vicinity of the Dirac-like point in the band structure. We show that when the effective refraction index of the GMMs is positive, the plasmons travel forward in the MGPW with a positive group velocity (vg > 0, vp > 0). In contrast—for the negative refraction index GMM claddings—a negative group velocity of the fundamental mode (vg < 0, vp > 0) appears in the proposed waveguide structure when the core is sufficiently narrow. A forbidden band appears between the negative and positive group velocity regions, which is enhanced gradually as the width of the core increases. On the other hand, one can overcome this limitation and even make the forbidden band disappear by increasing the chemical potential difference between the nanodisks and the ambient graphene of the GMM claddings. The proposed structure offers a novel scheme of on-chip electromagnetic field and may find significant applications in the future high density plasmonic integrated circuit technique.

## 1. Introduction

Negative-index materials (NIMs)—a class of photonic structures with simultaneously negative effective dielectric constant and permeability [1,2]—provide novel prospects for manipulating light. The phase velocity of electromagnetic (EM) field in such NIMs is directed against the flow of energy (vg > 0, vp < 0), which is inconsistent with the point view of the ‘conventional’ optics. Since Sir John Pendry predicted the NIM-based superlens in 2000 [3], NIMs have attracted worldwide interest. There are several approaches to obtain NIMs, such as photonic crystals (PhCs) [4,5], transmission lines [6] and their optical analogues [7]. Photonic crystals with a negative refractive index are commonly implemented by periodically varying parameters such as the dielectric constant [8], and further enable the exquisite control of light propagation in integrated optical circuits. They also emulate advanced physics concepts. However, the band structure of the conventional dielectric-based photonic crystals is determined as long as the geometry structure is defined due to the fixed dispersion of the permittivity and permeability. Thus, the tunable band structure is impossible in this category of photonic crystals. Nevertheless, graphene plasmonic metamaterials, which consist of a continuous graphene monolayer with an array of periodic distribution of chemical potentials, overcome this limitation and demonstrate broadband turnability [9]. These electrostatically tunable periodic structures may be derived by the standard complementary metal oxide semiconductor (CMOS) technology and offer a practical method for on-chip electromagnetic field manipulation in nanoscale [9,10]. Moreover, I Silveiro et al. presented unprecedentedly large values of the energy-transfer rate between an optical emitter and a layer of periodically doped graphene [11]. A Vakil et al. reported a theoretical study showing that by designing and manipulating spatially inhomogeneous, nonuniform conductivity patterns across a flake of graphene, one can treat this material as a one-atom-thick metallic platform for infrared plasmonic metamaterials and transformation optical devices [12]. In particular, L. Xiong et al. fabricated a tunable photonic crystal for SPPs with a continuous graphene monolayer and employed low-temperature near-field optical microscopy to visualize both propagating and localized SPPs in their photonic crystals [13].

Fast light is characterized by a group velocity vg = c/ng exceeding the vacuum speed of light c or taking negative values, where ng is the group-velocity index [14]. When the group velocity exceeds c, the optical waves propagate through the ‘fast-light’ media earlier than with the same distance in vacuum [15]. Various pioneering work has been reported to demonstrate the optical waves propagating in so-called ‘fast-light’ media, by using anomalous dispersion near an absorption line, nonlinear and linear gain lines or tunneling barriers [16,17,18,19]. As a result of the fascinating optical properties, manipulating the group velocity of the EM field has been widely studied in many potential applications, such as robust storage, retrieval of light pulses and quantum mutual information of an entangled state, etc. [20,21,22,23,24,25,26,27]. However, the conventional ‘fast-light’ structures suffer narrow operation frequency band and nonlinear dispersion relation, which further limit the applications [28]. Consequently, the structures with broad frequencies and linear dispersion are highly desired.

In this work, we propose a monolayer graphene plasmonic waveguide (MGPW) made up of a graphene core embedded in the graphene plasmonic metamaterial(s) (GMMs) claddings. The GMMs are composed of graphene nanodisks arrays which are immersed in the identical sheet of graphene with different chemical potential. These arrays are arranged in a square-shaped lattice with a lattice constant of a. Negative equivalent refractive indices of the GMMs are obtained by appropriately designed the structure of the graphene plasmonic metamaterials. The plasmonic waveguide is constructed by the graphene core and GMM claddings. We explore the ‘fast’ plasmon effect in the plasmonic waveguide by modifying the width of the graphene core and the chemical potential difference between the graphene nanodisks and the ambient graphene. In contrast to the conventional materials ‘fast-light’ media, the proposed broadly tunable two-dimensional ‘fast’ plasmon device offers the practical on-chip plasmon manipulation, ultracompact footprint and two- dimensional integratively [12] and may find broad applications in the field of light-matter interaction and high-density plasmonic integrated circuits in the future.

## 2. Calculation Methods and the Models

**Figure 1:**
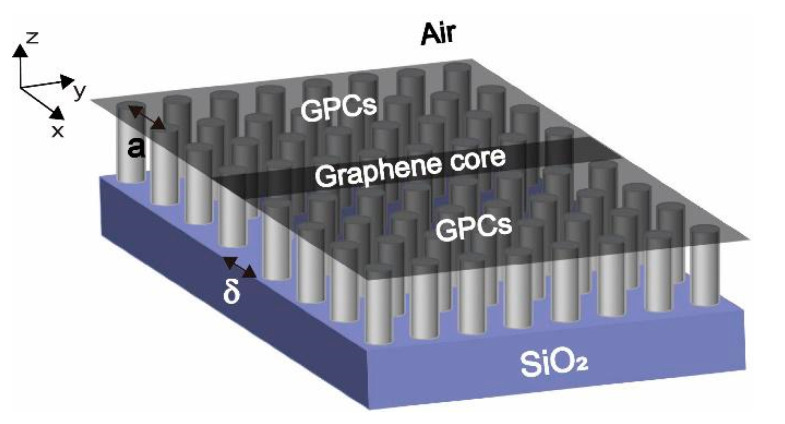
Schematic of the monolayer graphene plasmonic waveguide. From the XYZ coordinate system, a 3D structure, made from a SiO_2_ support, can be seen and then tall nanorods are grown on it, and finally there is a flat graphene sheet on the top.

As shown in Figure 1, the proposed waveguide is composed of a graphene core and graphene plasmonic metamaterial(s) (GMMs) claddings. The thickness of the graphene core is set as δ and the graphene plasmonic metamaterial(s) considered in this work is constructed by monolayer graphene, where two periodically arranged graphene nanodisks are surrounded by the same sheet of graphene with different chemical potential. The lattice constant of the graphene plasmonic metamaterials is a. During the possible fabrication process, silicon on insulator (SOI) is employed. Then, the negative tone electron beam resist is spun on the Si device layer, followed by electron beam lithography with the same pattern of the proposed graphene plasmonic waveguide structure. After development and hard baking, the remained pattern is utilized as the etching mask. Silicon column arrays with the same arrangement of the proposed structure are obtained by reactive ion etching (RIE) technique. Monolayer graphene is transferred onto the surface of the Si column arrays. Finally, external bias is applied between the graphene layer and the substrate. Periodical distribution of the chemical potential is achieved due to the periodical distribution of the dielectric constant under the whole graphene monolayer.

### 2.1. The Complex Refractive Index of a Graphene Sheet

Here, a monolayer of graphene sheet is placed on the x–y plane in Figure 1, below which is a substrate with a permittivity of $\;\underline{\varepsilon }$, and this sheet is immersed in a media with a permittivity of $\bar{\varepsilon }$. In the frequency region of interest, the graphene monolayer sheet behaves like a metal. Thus, the transverse magnetic (TM) polarized SPPs are therefore supported by the graphene sheet. The propagation constant of SPPs *q* is given by

(1) $$\begin{array}{c} q=\varepsilon_{0}\frac{\bar{\varepsilon }+\underline{\varepsilon }}{2}\frac{2i\omega }{\sigma_{g}} \end{array}$$

where $\bar{\varepsilon }$ = 1 and $\underline{\varepsilon }$= 3.9 represent the dielectric constants of super and substrates, ε_0_ is the permittivity of the free space. According to the Kubo formula, the surface conductivity of graphene is composed of contributions from intraband electron–phonon scattering *σ_intra_* and interband electron transitions *σ_inter_*, i.e., $\sigma_{g}\left(\omega \right)=\sigma_{intra}+\sigma_{inter}$

(2) $$\begin{array}{c} \sigma_{intra}=\frac{ie^{2}k_{B}T}{\pi \hbar^{2}\left(\omega +i\tau^{-1}\right)}\left[\frac{\mu_{c}}{k_{B}T}+2ln\left(1+\exp\left(-\frac{\mu_{c}}{k_{B}T}\right)\right)\right] \end{array}$$

(3) $$\begin{array}{c} \sigma_{inter}=\frac{ie^{2}}{4\pi \hbar }ln\left(\frac{2\left|\mu_{c}\right|-\left(\omega +i\tau^{-1}\right)}{2\left|\mu_{c}\right|+\left(\omega +i\tau^{-1}\right)}\right) \end{array}$$

where *e* is the electron charge, ℏ denotes the reduced Planck constant and kB represents the Boltzmann’s constant, *T* = 300 K is the temperature, *μ_c_* is chemical potential, *ω* is the angular frequency. The momentum relaxation time is chosen as *τ* = 1 ps. Utilizing Equation (2), we obtain the complex effective refractive index of SPPs $N=\frac{q}{k_{0}}$. Thus, the optical response of graphene is characterized by its surface conductivity which is further controlled by the chemical potential. The chemical potential of graphene can be effectively tuned via chemical doping or an external gate voltage. It should be noted that the effect of absorption and radiation losses k is characterized by the imaginary part of N, which is much smaller than the real part.

### 2.2. The Effective Permittivity, Permeability of the Graphene Metamaterial with Square Lattice

**Figure 2:**
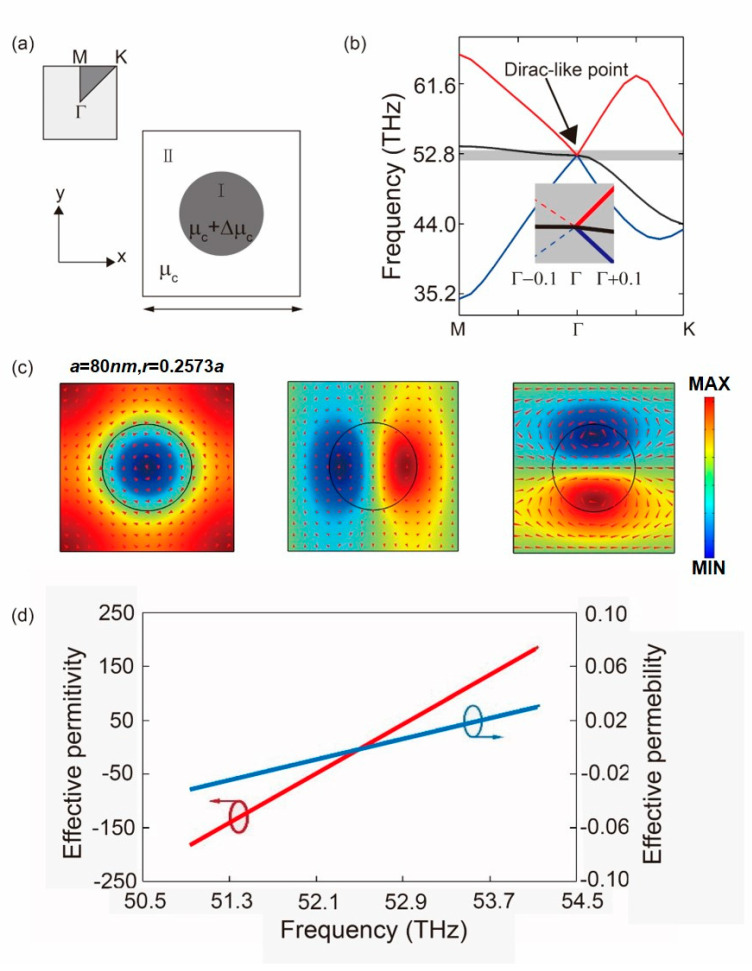
Schematic of the graphene metamaterials (GMMs) and band structure. (**a**) Proposed graphene metamaterials (GMMs) in real and reciprocal space. The chemical potential of the graphene nanodisk (region I) and the ambient graphene (region II) are $\mu_{c}+∆\mu_{c}$ and $\mu_{c},$ respectively and the Brillouin zone corresponding to the square-shaped unit cell is placed in the upper left corner; (**b**) The band structure of the proposed GMMs and the illustration is the detailed information around the Dirac-like point; (**c**) The eigen electric fields including two dipolar modes and a single monopole mode the arrow indicate the eigen magnetic fields; (**d**) effective permittivity and permeability of the GMMs.

The proposed graphene metamaterial is shown in Figure 2a, the ambient (region I) is monolayer graphene with a chemical potential of $\mu_{c}$ = 0.6 eV. $∆\mu_{c}\left(x,\;y\right)$ is the ‘relative chemical potential’, i.e., the deviation from ambient chemical potential. The band structure of the proposed plasmonic crystal is calculated by using the commercially available finite element method (FEM)-based software, COMSOL Multiphysics, RF module. As shown in Figure 2b, when $∆\mu_{c}$ = −0.3 eV, there is a critical point (*r* = 0.2573*a*), where triple accidental degeneracy of the electromagnetic field takes place at the Brillouin zone center (Γ) which is known as the Dirac-like point. Figure 2b displays the detailed information around the Dirac-like point. For an individual unit cell, each photon frequency ω(*k*_x_, *k*_y_) in Figure 2b supports eigenstates (eigen electric/magnetic fields). At the Dirac-like point, the eigen electric fields $E_{z}^{\left(\Gamma \right)}\;\left(r\right)$ including two dipolar modes and a single monopole mode are plotted in Figure 2 and the arrows indicate the direction of the eigen magnetic fields $H_{x}^{\left(\Gamma \right)}\left(r\right)$, where r = (x, y) is the coordinate in real space. In order to calculate the effective permittivity, permeability of the GMMs at the vicinity of the Dirac-like point, we treat GMMs as a homogenous medium, which is confirmed by the 3-dimensional Dirac-like cone-band structure. Hence the SPPs sustained by GMMs are assumed to be plane waves with fixed wave vector ***k*** for simplicity. At a given frequency, the electric (magnetic) field envelope functions of SPPs, $\bar{E_{z}}\;\left(\bar{H_{x}}\right)$, can be simply treated as constants. Considering the geometry of an individual unit cell divided into two areas (I, II in Figure 2a), we get the field envelope functions [27]:

(4) $$\bar{E_{z}}=\frac{\sqrt{\iint_{I}E_{z}^{\left(k\right)}\left(r\right)dxdy}\sqrt{\iint_{\mathrm{II}}E_{z}^{\left(k\right)}\left(r\right)dxdy}}{\xi }$$

(5) $$\bar{H_{x}}=\frac{\sqrt{\iint_{I}H_{x}^{\left(k\right)}\left(r\right)dxdy}\sqrt{\iint_{\mathrm{II}}H_{x}^{\left(k\right)}\left(r\right)dxdy}}{\xi }$$

where $\xi =\sqrt{S_{I}}\sqrt{S_{\mathrm{II}}}$ denotes the geometric average of all the areas. When ω is close to the Dirac-like point, the wave vector of SPPs in GMMs, $k\;=\;\left(0,\;k_{y}\right)$, varies along the *k*_y_ direction. The effective permittivity, permeability of the s reduce to $\varepsilon_{GMMs}\left(\omega \right)=k_{y}\bar{E_{z}}/\left(\omega \varepsilon_{0}\bar{H_{x}}\right)$, $\mu_{GMMs}\left(\omega \right)=k_{y}\bar{H_{x}}/\left(\omega \mu_{0}\bar{E_{z}}\right)$ by substituting the envelope functions into Maxwell’s equation. As shown in Figure 2c, when the frequency of the propagation SPPs is smaller than 52.6 THz, the effective permittivity and permeability are simultaneously negative, resulting in negative refraction index of the graphene plasmonic claddings. As shown in Figure 2d, both the effective permittivity and permeability pass through zero and linearly.

### 2.3. The Monolayer Graphene Plasmonic Waveguide and Dispersion Relation

**Figure 3:**
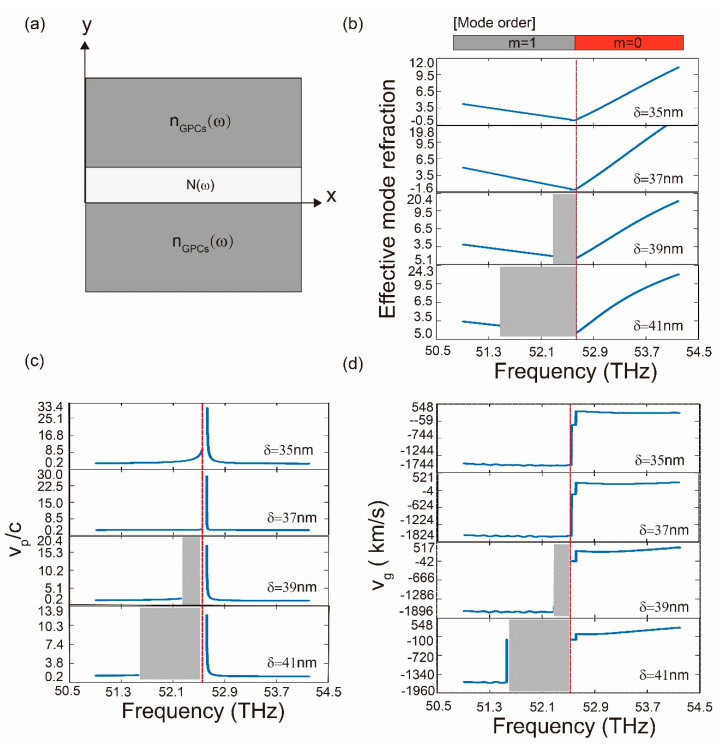
Schematic of a monolayer graphene plasmonic waveguide and the dispersion relation of the SPPs modes in the monolayer graphene plasmonic waveguide (MGPW). (**a**) Schematic of monolayer graphene plasmonic waveguide to calculate the dispersion relation; (**b**) effective mode refraction of the SPPs modes in the MGPW; (**c**) phase velocity of the SPPs modes in the MGPW; (**d**) group velocity of the SPPs modes in the MGPW.

In this section, we consider a situation that SPPs travel through the monolayer graphene plasmonic waveguide composed of the graphene core (complex refraction N=n+ik) sandwiched by GMMs claddings (effective refraction index $n_{GMMs}\left(\omega \right)=\sqrt{\varepsilon_{GMMs}}\sqrt{\mu_{GMMs}}$), as shown in Figure 3a. The dispersion relation for TM polarized SPPs of this proposed structure is given by [28]:

(6) $$\begin{array}{c} \gamma {\left(1-ℛ\right)}^{\frac{1}{2}}=2\tan^{-1}\left[\frac{1}{\mu_{GMMs}\left(\omega \right)}{\left(\frac{ℛ}{1+ℛ}\right)}^{\frac{1}{2}}\right]+m\pi \end{array}$$

where *m* is the mode order. ℛ = (n*_eff_*^2^(ω) − n*_GMMs_*^2^(ω))/(n(ω)^2^ − n*_GMMs_*^2^(ω)) represents the normalized refraction index with the effective mode refraction written as n*_eff_*(ω) = β /*k*_0_. When n*_GMMs_*(ω) > n(ω), the normalized frequency γ = *k*_0_δ$\sqrt{n_{GMMs}{\left(\omega \right)}^{2}-n{\left(\omega \right)}^{2}}$. The width of graphene core, δ, enables the texturing the normalized frequency range, whereas the narrower the width of graphene core, the smaller the normalized frequency. Therefore, one can effectively observe the particular SPPs modes in the MGPW by varying the width of the graphene core.

In addition, according to the Equation (6), frequency and graphene’s chemical potential determine the refractive index difference between the core and claddings $n_{GMMs}{\left(\omega \right)}^{2}-n{\left(\omega \right)}^{2}$. The normalized frequency γ depends on the thickness of the graphene core and the refractive index difference and the normalized refractive index ℛ depends on the refractive index difference. The normalized frequency and the normalized refractive index constitute the dispersion equation. Therefore, the study can be divided into two parts. One part is to fix the refractive index difference (depending on the chemical potential and frequency) and change the thickness of the core layer. The second part is to fix the thickness of graphene core and change the refractive index difference.

## 3. Results and Discussions

### 3.1. Effective Mode Index, Group and Phase Velocities of the Proposed Waveguide

To demonstrate the negative group velocity of the plasmon, an incident SPPs is launched at the input of the monolayer graphene plasmonic waveguide with the eigen mode. The chemical potential of the ambient graphene is $\mu_{c}$ = 0.6 eV. The chemical potential difference between the graphene nanodisks and the ambient graphene is set as $∆\mu_{c}$ = −0.3 eV. The radius of the graphene nanodisks is *r* = 0.2573*a*, which allows us to get the Dirac-like point in the band structure. In Figure 3b, we show the dispersion relation (*n_eff_* versus ω) for three widths of graphene core of in two different cases, where the graphene core is embedded in the positive index and negative index claddings, respectively. For the MGPW with positive index claddings, the effective mode refraction increases in proportion to the frequency ω when *m* = 1. Therefore, it corresponds to the normal dispersion. In contrast, in the case of the MGPW with negative index claddings, there exist narrow spectral regions where *n_eff_*(ω) is a decreasing function of frequency (that is *dn_eff_*/*d* ω < 0), which is known as anomalous dispersion. It should be noted that the mode order *m* takes zero, which corresponds to the fundamental SPPs mode traveling through the plasmonic structure. In addition, the phase velocity of the SPPs sustained by the MGPW is characterized by the *v_p_* = ω/β, which is plotted in Figure 3c, as a function of photon frequency ω. In this figure, when the frequency ranges from 50.7 to 52.6 THz, the effective refraction index of GMMs takes negative value and the phase velocity of SPPs increase gradually. However, when the SPPs range in frequency from 52.6 to 54.3 THz, the phase velocity significantly decreases. Furthermore, the group velocity is simply written as *v_g_* = ∂ ω /∂β = c/n*_g_*, where n*_g_* is the group-velocity index. For the MGPW of length *L*, it takes a propagation time *L*/v*_g_* = n*_g_L*/*c* for SPPs modes. The SPPs modes that enter the proposed MGPW possess at a moment delayed by a time difference ∆T = *L*/*v_g_*-*L*/*c* = (*n_g_* − 1)*L*/*c*, where *L*/*c* denotes the vacuum transit time. As shown in Figure 3d, the group velocity takes positive values when the frequency ranges from 52.6 to 54.3 THz. In contrast, at the frequency ranging from 50.7 to 52.6 THz, the group velocity and the phase velocity are antiparallel to each other when *m* = 0. The delay −∆T = (1−*n_g_*)*L*/*c* becomes larger than the *L*/*c*, meaning that the backward fundamental SPPs mode (fast light) appears with the anomalous dispersion.

**Figure 4:**
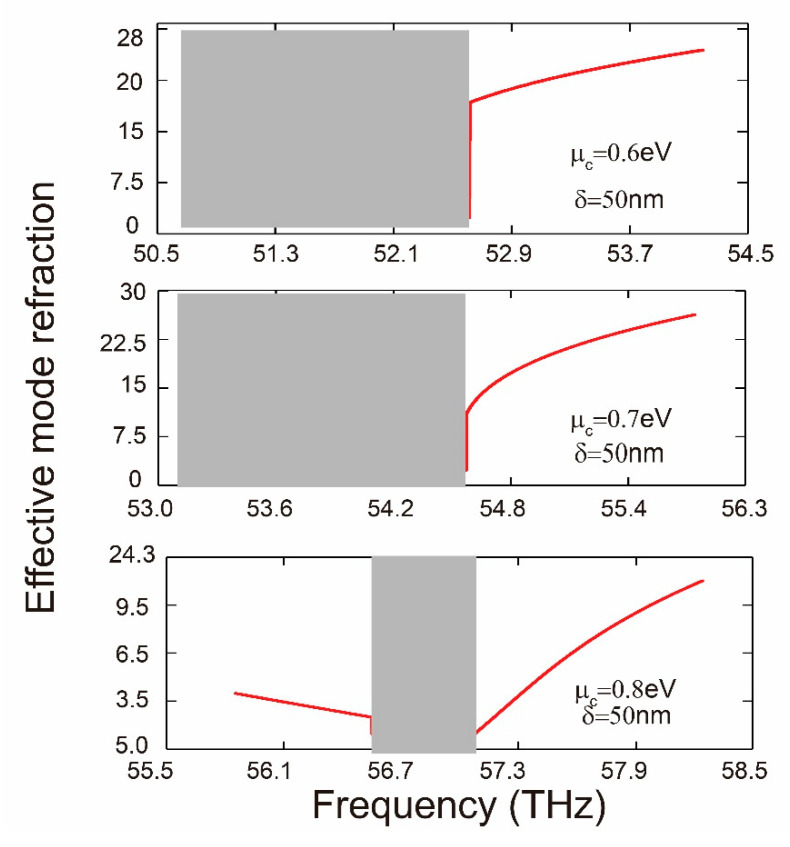
Effective mode refraction for the MGPW as a function of frequency with different chemical potentials in the core.

It is important to point out that, as shown in Figure 3a–c, for the configuration at the critical point (δ = 37 nm), the backward fundamental SPP modes exist with the frequency ranges between 52.6 and 54.3 THz. As described in the introduction, “fast” plasmons is a physical phenomenon, where the group velocity $v_{g}$ of optical pulses faster than c, the speed of light in vacuum—or even takes on negative value. Backward waves in this paper possess negative group velocity and called fast light. In addition, for the thickness of graphene core δ > 37 nm, a forbidden band starts to appear and gradually enlarges, meaning that the backward fundamental SPPs mode steadily vanishes as the width of the graphene core increasing. In order to increase the critical thickness δ and narrow down this forbidden band, we demonstrate that modifying the chemical potential enhances the fundamental SPPs mode. As shown in Figure 4, when we increase the difference of the chemical potential between the nanodisks and the ambient $∆\mu_{c}$, while keeping the chemical potential of graphene nanodisks and the width of graphene core (δ = 50 nm) constant, the width of the forbidden band decreases.

### 3.2. Numeric Demonstration of the Fast Plasmon Propagating in the MGPW with Graphene Core and Negative-Index Claddings

**Figure 5:**
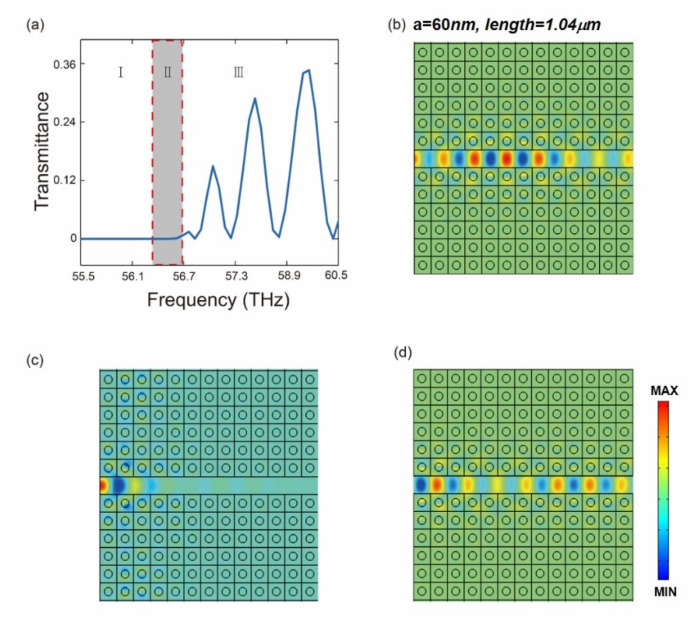
Distinction between backward propagation, forbidden band and forward propagation. (**a**) Transmittance of backward propagation, forbidden band and forward propagation; (**b**–**d**) are the light field of backward propagation, forbidden band and forward propagation, respectively. The pitch of the metamaterial in (**a**–**c**) is 1040 nm×770 nm.

We numerically investigate the backward (forward) fundamental SPPs in the MGPW with negative(positive) index GMMs claddings. The lattice constant of the GMMs is *a* = 60 nm. The chemical potential of the ambient graphene is $\mu_{c}$ = 0.8 eV. The difference of the chemical potential between the graphene nanodisks and the ambient graphene is $∆\mu_{c}$ = − 0.5 eV. The disk radius *r* = 0.2023*a*. The width of the graphene core is δ = 50 nm. To compare the negative and the positive group velocities of the SPPs in the proposed MGPW, we extend the scanning frequency ranged from 55.5 to 60.5 THz. The transmission spectrum is plotted in Figure 5a, which is divided into three parts, part I, where the frequency ranges from 55.5 to 56.5 THz, belongs to negative group velocity region, part II, where the frequency ranges from 56.5 THz to 56.7, belongs to propagation forbidden region and part, III where the frequency ranges from 56.7 THz to 60.5 THz, belongs to positive group velocity region. It is clear that the transmission in part I and the part II is ultralow, which is due to the fact of the backward propagation nature in part I and the forbidden nature in part II. However, the transmission is enhanced in part III, where the group velocity is positive, i.e., it is the forward propagating wave. Figure 5b–d show the typical SPPs field distribution of the three regions, respectively. There is SPPs field distribution in Figure 5b, though the transmission is almost zero. This is due to the fact of backward propagation. No noticeable light field distribution is found to pass through the waveguide in Figure 5c, since it is in the region of forbidden propagation. SPPs field distribution shown in Figure 5d and the transmission curve shown in part III in Figure 5a further confirms the fact of positive velocity. Note that the oscillation in part II may due to the Fabry–Pérot resonance [29,30]. Consequently, the SPPs field distribution resulting from the FEM technique agree well with the results of the dispersion relation equations when effective refractive index the claddings of the MGPW is negative and positive, respectively. The dynamic diagram of SPPs fields of backward and forward propagation are exhibited in the Supplementary Materials Videos S1 and S2.

### 3.3. Comparison Between the Results from Gaussian Pulse Propagation and Analytical Dispersion Relation

**Figure 6:**
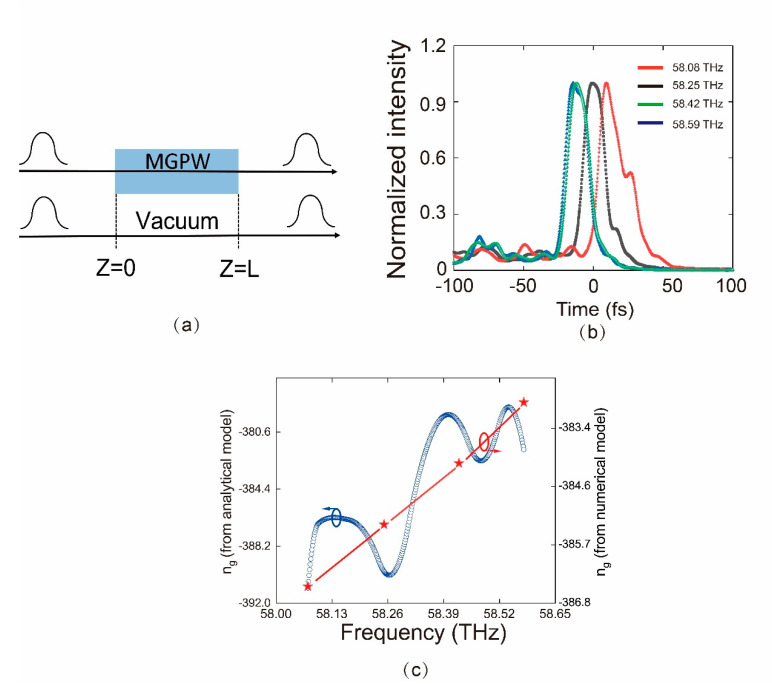
Distinction between backward propagation, forbidden band and forward propagation. (**a**) Transmittance of backward propagation, forbidden band and forward propagation; (**b**–**d**) are the light field of backward propagation, forbidden band and forward propagation, respectively. The pitch of the metamaterial in (**a**–**c**) is 1040 nm×770 nm.

To further verify the negative group velocity nature of the plasmon, we investigate the propagation of the Gaussian pulses through the proposed waveguide illustrated in Figure 6a. The incident Gaussian probe pulses with 85 *fs* of full width at half maximum (FWHM), are modulated by sinusoidal carrier waves at the entrance of the MGPW (*z* = 0) and recorded numerically at the rear of the MGPW (*z = L*). For comparison, the identical light pulses traversing in vacuum with the same distance are also numerically recorded. Because the chemical potential difference between the nanodisks and the ambient graphene limits the “fast” plasmons propagating, we use a relative chemical potential of $∆\mu_{c}\;$= −0.6 eV to avoid the forbidden band and hence to optimize the anomalous dispersion. The thickness of the graphene core is set as 50 nm. For the carrier frequency ranging from 58.08 to 58.59 THz, the effective refractive index of the GMMs is negative and essentially linear with respect to the frequency. Figure 6b shows the envelope of the modulated SPPs pulses propagating through the 20-μm length of the MGPW with negative group velocities. We calculate the group-velocity index n*_g_* of the SPPs Gaussian pulses, which is proportional to the angular frequency. The results are illustrated in Figure 6c. In addition, the analytical group n*_g_*—which is obtained from the analytical Equation (6)—is included in the same diagram. It should be noted the group-velocity index n*_g_* obtained from the two methods are consistent with each other in general. The analytical n and the fluctuation of the analytical curve may be resulted from the interpolation to the effective mode refraction.

Comparing with other reported structures of negative group velocity [16,17,18,19], our structure offers advantages such as ultra-compact footprint, flexible design and control of the device structure and suitable for two dimensional integration. Practically, the electromagnetic response of our proposed structure may be detected by the approach reported by L. Xiong, D.N. Basov and coworkers [13]. Cryogenic near-field imaging techniques can be employed to visualize both propagating and localized SPPs in our monolayer graphene plasmonic waveguide. First, a metallic tip of an atomic force microscope (AFM) is illuminated by focused IR radiation from a laser source. Then, the tip acts as an optical antenna which resonantly enhances the electric field at the apex. The tip-scattered light is registered by a detector and the amplitude and phase of the corresponding near-field signal are extracted by a proper demodulation scheme.

## 4. Conclusions

In conclusion, we propose a monolayer graphene plasmonic waveguide composed of the graphene core and metamaterial claddings. Emulating the propagation waves propagating through the dielectric three-layer waveguide, this structure supports SPPs modes that can be confined in the graphene core. When the refraction index of the GMM takes positive values, the proposed MGPW supports the forward fundamental SPPs mode. In contrast, the MGPW with negative index supports the backward fundamental SPPs mode when the width of graphene is narrow enough. Additionally, a forbidden band lies between the positive and negative group velocity regions. Furthermore, forbidden band gradually enlarges with the width of graphene core. Our proposed structure may offer a novel approach to manipulate electromagnetic fields in nanoscale.

## Supplementary Materials

The following are available online at https://www.mdpi.com/2079-4991/10/9/1637/s1, Video S1: The backward propagation, Video S2: The forward propagation.
