# Supplementary figures and images for: “Fast” Plasmons Propagating in Graphene Plasmonic Waveguides with Negative Index Metamaterial Claddings
Source: Nanomaterials (Basel). 2020 Aug 20;10(9):1637. doi: 10.3390/nano10091637 (PMC7557730; doi:10.3390/nano10091637)

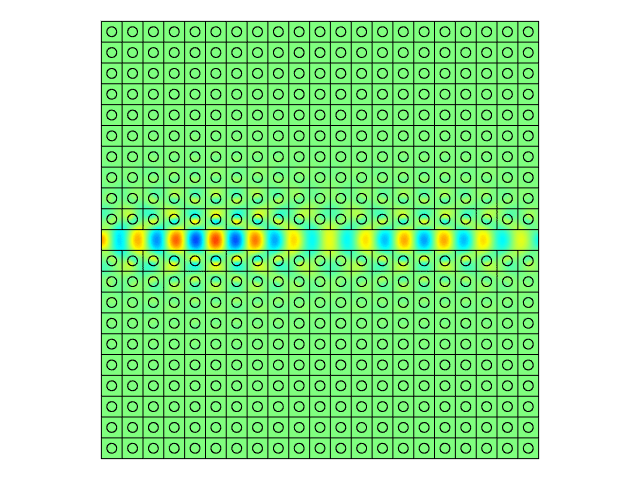

Supplement: Supplementary file 1 [file nanomaterials-10-01637-s001.zip › forward propagation.gif]

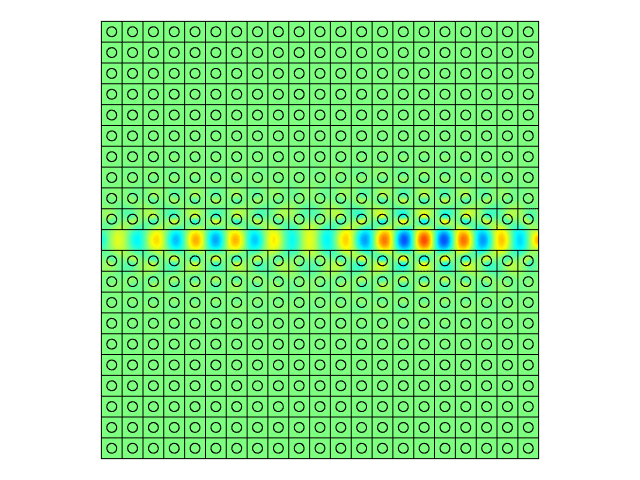

Supplement: Supplementary file 1 [file nanomaterials-10-01637-s001.zip › backward_propagation.gif]
